# A roadmap of artificial intelligence applications in pediatric surgery: a comprehensive review of applications, challenges, and ethical considerations

**DOI:** 10.1007/s00383-025-06185-6

**Published:** 2025-09-06

**Authors:** Miriam Duci, Arianna Bossi, Francesca Uccheddu, Francesco Fascetti-Leon

**Affiliations:** 1 Department of Women’s and Children’s Health, University of Padova, Padua, Italy; 2 Pediatric Surgery Unit, Division of Women’s and Children’s Health, Padova University Hospital, Padua, Italy; 3 Department of Industrial Engineering, Padova University, Padua, Italy; 4 Division of Pediatric Surgery, Department of Women’s and Children’s Health, University of Padova, Via Giustiniani 2, 35128 Padua, Italy

**Keywords:** Aritficial intelligence, Machine learning, Computer vision, Natural language processing, Pediatric surgery

## Abstract

Artificial intelligence (AI) and machine learning (ML) are rapidly transforming healthcare, with growing interest in their application to rare pediatric surgical conditions. In these settings, limited data availability often brakes traditional research. Although pediatric surgery has historically been slower than other specialties in adopting ML, recent years have seen an increase in AI-driven tools designed for surgical care. This review presents an overview of AI applications in pediatric surgery, highlighting current uses, benefits, challenges, and their potential clinical impact. A comprehensive literature search was conducted to identify studies on AI and ML models relevant to pediatric surgery. The findings indicate that ML is mainly applied in predictive decision support, particularly for preoperative risk stratification, intraoperative navigation, and postoperative outcome prediction. AI is especially valuable in endoscopic and minimally invasive procedures, where it enhances the visualization of anatomical landmarks. In pediatric oncologic surgery, AI aids in the accurate localization and delineation of tumors. Additionally, AI improves pathology workflows through automated image analysis and annotation, supporting both diagnosis and education. Despite these advances, ethical and regulatory challenges remain. Ensuring data privacy and obtaining informed consent are essential. When responsibly implemented, AI can significantly improve pediatric surgical care.

## Introduction

Artificial intelligence (AI) and machine learning technologies are rapidly transforming nearly all fields, including health-care. The entire medical scientific community is fascinated by the opportunity to apply these algorithms in clinical practice, especially in addressing rare conditions to overcome the scarcity of data in existing series, facilitating a more robust and supported diagnostic and therapeutic process [1]. Although some sub-fields of medicine, such as pediatric surgery, have been relatively slow to obtain the critical benefits of deep learning, related research in this field is begging to accumulate significantly [2]. Hence, in this paper, we examined recent AI applications relevant to pediatric surgery. This review aimed first to provide an overview of current AI implementations, highlighting specific machine learning/AI limitations in pediatric surgery field and second to categorize pediatric surgery-related AI applications into macrodomains, to explain their sub-domains and the important elements of the applicable AI models discussing how these technologies will advance the field of pediatric care.

## Methods

PubMed was searched from database inception to January 15 2025, for articles addressing AI in pediatric surgery. To guide our search, the search terms of “pediatric surgery”, “artificial intelligence”, “machine learning”, “deep-learning”, “computer vision” and “natural language processing” were used combined with Boolean operators “AND”, “OR”. Articles written in languages other than English and articles in the form of conference abstracts, editorials, and commentaries were excluded. There was no further limitation on study design. After the selection of studies, data from each article were extracted and organised into 3 main subset of AI (Machine learning, computer vision, Natural language processing) in a standardised data extraction form developed in Microsoft Excel. This was done independently by two authors to record the information and synthesise it in summary format (M.D, A.B). Extracted information included author name(s), year of publication, title, location of study, study design, goal of study, target study population, description of discussed AI intervention, domain of AI used, data source, evaluations of AI tool accuracy/efficacy, main results of the study, identified barriers to clinical integration of described application were extracted.

## Results

Our comprehensive review identified a wide range of artificial intelligence (AI) subsets currently explored within pediatric surgery, each offering unique strengths and applications. Among these, the most prominent are Machine Learning (ML), Computer Vision, and Natural Language Processing (NLP).

### Machine learning

Machine learning, a subset of AI, is a scientific discipline focused on understanding how computers can learn from data. It is broadly defined as the computational ability to learn from experience, recognize patterns, and make predictions. In recent years, ML has emerged as a cornerstone of AI applications in medicine, including pediatric surgery, where it plays a key role in improving diagnosis, optimizing treatment decisions, and predicting outcomes [3]. ML encompasses a variety of algorithms designed to classify data accurately or make precise predictions based on the analysis of structured and unstructured data. It is generally categorized into three main types: supervised, unsupervised and reinforcement learning (Table 1). In pediatric surgery, ML is most commonly used to develop tailored clinical decision support systems and predictive models for surgical outcomes, complications, and patient survival.

**Table 1:** Definition of machine learning approach

Supervised learning:	Learns from labeled medical data with known outcomes (for instance Reisman et al. [7])
Unsupervised learning	Discovers patterns in unlabeled medical data without predefined targets. (for instance, Sylvester et al. [18])
Reinforcement learning	Learns to make sequential decisions by interacting with an environment and improving based on feedback

#### Clinical Decision Support Systems (CDSS)

We consider the most common area where ML has been applied in the pediatric surgery filed.

##### Appendicitis diagnosis and management

Recent advancements in ML have significantly improved the diagnosis and management of appendicitis, the most common emergency in pediatric surgery. The studies used a variety of ML approaches, considering data from clinical evaluations, laboratory measurements, and diagnostic imaging to create and assess clinical diagnostic tools.

AI solutions offer similar sensitivity and superior specificity to existing scoring systems, like idelberg Appendicitis Score (HAS), Pediatric Appendicitis Score (PAS), Alvarado-Score, and Tzanakis-Score. AI tools can integrate multifaceted data inputs to provide more precise risk stratification and diagnostic predictions [4, 5]. Specifically Aydin et al. developed a decision tree model based on clinical, laboratory, and radiological findings, which achieved a 94% AUC and 95% accuracy for appendicitis diagnosis. However, the model's performance dropped when distinguishing complicated cases (AUC 79%, accuracy 70%) [6]. Similarly, Reisman et al. applied supervised learning, incorporating ultrasound-based appendix diameter, achieving AUCs of 0.91 to diagnose appendicitis [7]. Marcinkevics et al. based on a range of information encompassing history, clinical examination, laboratory parameters, and abdominal ultrasonography, compared multiple ML models and identified random forests as superior, with AUCs of 0.94 (diagnosis), 0.92 (management), and 0.70 (severity) [8]. Their findings led to the development of a user-friendly online prediction tool. Despite the current lack of generalizability, each of the studies reviewed showed encouraging findings for the use of AI to diagnose appendicitis in pediatric population.

##### Hirschsprung disease (HD)

ML techniques have shown significant promise in improving the diagnosis of HD. Traditional diagnostic methods, such as contrast enemas and histological assessment of rectal biopsies, can be subjective and require specialized expertise. ML-based approaches are helping to overcome these challenges. For instance, Huang et al. developed support vector machine (SVM) and logistic regression models that outperformed radiologists in identifying short-segment HD, achieving AUCs of 0.91 and 0.93, respectively [9]. In the histopathological domain, Duci et al. applied a U-Net convolutional neural network (CNN) to automatically detect ganglion cells and hypertrophic nerves on digital histology slides, reaching impressive accuracies of 92.3% and 91.5% [10]. Similarly, Greenberg et al. applied hierarchical contextual analysis (HCA) to histological data, achieving 96% sensitivity and 99% specificity for ganglion cell detection, with multi-center validation confirming 99.2% classification accuracy [11]. These advances in deep learning and feature-based classification have the potential to assist pathologists in challenging cases, reduce diagnostic delays, and standardize interpretation across centers. As these tools continue to evolve, they may be integrated into digital pathology workflows, especially in resource-limited settings, to support earlier and more reliable HD diagnosis.

##### Necrotizing enterocolitis (NEC)

NEC remains a life-threatening condition in neonates, and ML has been extensively used to identify early biomarkers and stratify disease severity. In a pioneering study, Mueller et al. used artificial neural networks (ANN) on 57 clinical variables and identified small for gestational age and mechanical ventilation as key predictors of NEC [12]. For biomarker discovery, Pantalone et al. employed random forest (RF) algorithms to analyze complete blood count (CBC) data at different time points before NEC onset, distinguishing effectively surgical NEC from controls but was less successful in differentiating surgical from medical NEC [13]. Six supervised ML models on 74 clinical variables were explored with logistic regression (LR) and RF as the best models identifying key predictors such as gestational age, birth weight, maternal chorioamnionitis, surfactant and patent ductus arteriosus therapy (medical or surgical ligation), as key differentiators for NEC prediction accuracy [14]. In addition, a recent model combined ResNet34 (for imaging) and one-dimensional CNN (for lab data) across 408 abdominal X-rays and 11,016 lab tests. The model achieved 94% accuracy and an AUC of 0.91, outperforming traditional methods and matching expert-level diagnostic accuracy [15]. Recent advancements have also explored the combination of ML techniques with non-invasive monitoring tools such as near-infrared spectroscopy (NIRS) to improve NEC risk assessment. Verhoeven et al. integrated ML with NIRS data to detect low abdominal oxygen saturation (ArSO₂ < 50%) within 24 h of life—linked to a higher NEC incidence. ML models analyzing continuous NIRS monitoring could aid in early NEC detection and intervention. ML algorithms have also demonstrated significant promise in stratifying NEC severity [16]. Ji et al. introduced an ML-based risk stratification model using linear discriminant analysis (LDA) on features such as pneumatosis intestinalis, portal venous gas, and metabolic acidosis at the onset of the disease [17]. Their model predicted NEC severity with an AUC of 0.85 and showed strong agreement with manual staging. Sylvester et al. combined urine peptide profiling with unsupervised ML to distinguish surgical from medical NEC [18]. Their integrated LDA model outperformed clinical-only models in stratifying NEC severity. More recently, a novel Ridge Regression and Q-learning-based Bee Swarm Optimization (RQBSO) algorithm, a hybrid approach offering robust optimization capabilities in AI-driven healthcare, was applied to identify key clinical and laboratory parameters as significant indicators of severe NEC requiring surgery. Their model identified key features like anemia, high WBC, peritoneal signs, and early onset as severe predictive factors achieving 91.88% AUROC [19].

##### Pediatric urology

ML is increasingly being applied in pediatric urology to enhance diagnostic precision and support clinical decision-making, particularly in conditions where interpretation of imaging or anatomical classification can be complex. Blum et al. developed a supervised learning algorithm that analyzed features extracted from diuresis renograms to identify ureteropelvic junction obstruction (UPJO), achieving an impressive area under the curve (AUC) of 0.96. This approach demonstrates the potential for ML to reduce diagnostic subjectivity and streamline the evaluation of functional uropathies [20]. Further extending ML applications to urinary tract conditions, Babajide et al. repurposed a brain MRI segmentation deep learning model to accurately characterize urinary stones on CT in both adult and pediatric patients, achieving high sensitivity and specificity while outperforming radiologists in measurement efficiency [21]. For vesicoureteral reflux (VUR), Khonder et al. used voiding cystourethrogram features to train a random forest model (qVUR), improving grading reliability over expert consensus [22]. Kabir et al. further refined this approach by incorporating additional anatomical features to enhance discrimination between grades 3 and 4 [23]. Similarly, Sloan et al. applied radiomic texture analysis to ultrasound images, creating a support vector machine model to classify hydronephrosis severity, achieving an AUC of 0.86 [24]. In another innovative application, Fernandez et al. trained a convolutional neural network (CNN) to classify images of hypospadias. The model improved diagnostic accuracy from 75 to 90% through iterative learning, ultimately approaching the level of agreement observed among expert clinicians [25].These developments highlight how ML tools can support non-invasive, image-based diagnostics and standardize evaluations in pediatric urology, offering substantial benefits for early detection, classification, and treatment planning.

#### Predictive models

Machine learning has emerged as a powerful tool in precision medicine, particularly for predicting long-term outcomes and complications in pediatric surgical patients. By analyzing complex, multidimensional data, ML algorithms can uncover patterns beyond the reach of traditional statistical methods. We consider the most common area where ML has been applied in the pediatric surgery filed.

##### Postoperative monitoring in appendicitis

Two studies focused on predicting complications after appendectomy. Ghomrawi, H. M. K combined clinical, demographic, and wearable device (Fitbit) data to train a random forest model aimed at early detection of post-operative complications. This model accurately detected 83% of these abnormal recovery days in complicated appendicitis and 70% of abnormal recovery days in simple appendicitis prior to the true report of a symptom/complication, supporting the development of machine learning algorithms to predict onset of abnormal symptoms and complications in children undergoing surgery, and the use of consumer wearables as monitoring tools for early detection of postoperative events [26]. Similary, Eickhoff et al. used a 10-year retrospective dataset to build a model predicting Intensive Care Unit (ICU) admission and prolonged stay in children with perforated appendicitis. Their model achieved up to 88% accuracy for ICU duration prediction (sensitivity and specificity 88%) and 68% accuracy for complications in new cases based on demographic and surgical baseline characteristics [27].

##### Neonatal postoperative mortality

Cooper et al. developed a Super Learner ensemble algorithm to predict 30-day postoperative mortality in neonates, leveraging comprehensive preoperative data—including patient demographics, clinical characteristics, and indicator variables for surgical procedures [28]. By integrating multiple candidate models, the algorithm demonstrated strong predictive performance, achieving an AUROC of 0.91 in the development cohort and 0.87 in the validation cohort, performing comparably to established risk assessment tools. More recently, tree-based ensemble models, particularly Random Forest (RF) and XGBoost, have shown superior performance in predicting adverse outcomes among preterm infants. Chi-Hung Shu et al. used 47 maternal and neonatal clinical variables, encompassing maternal and neonatal characteristics, medication and pregnancy history, as well as neonatal interventions, treatments, and conditions in the NICU. The AUROC values for bronchopulmonary dysplasia (BPD), NEC, sepsis (with or without meningitis), and mortality all exceeded 0.7, indicating fair predictive power. However, the area under the precision-recall curve (AUPRC) values for each outcome surpassed the respective prevalence rates, highlighting the models’ ability to accurately identify true positive cases among very low birth weight (VLBW) preterm infants [29].

##### Pediatric oncology

In pediatric oncology, Chen et al. applied ML techniques to predict five-year survival in patients with Ewing sarcoma using the Surveillance, Epidemiology, and End Results database (SEER). They evaluated four algorithms—boosted decision tree, support vector machine, random forest, and neural network—with random forest emerging as the top performer. The model achieved an AUC of 0.91 for cancer-specific survival and 0.94 for overall survival [30]. These findings align with broader efforts in pediatric oncology to leverage machine learning for prognostication. For instance, Gurumurthy, G et al. demonstrated that ML-based prediction models could accurately estimate long-term outcomes in pediatric patients with various malignancies, improving clinical decision-making and individualized care planning [31].

### Computer vision

Computer vision enables machines to interpret and analyze visual data, emulating human vision. In pediatric surgery, CV is transforming clinical practice by enhancing diagnostic accuracy, improving surgical planning, and enabling real-time intraoperative assistance. From image segmentation to surgical navigation, CV applications are increasingly integral to optimizing patient care. Key developments include:

#### Image segmentation and diagnostic support

Image segmentation enables automated systems to detect and delineate anatomical structures or pathological features within medical images. In pediatric oncology, this capability enhances tumor localization while preserving adjacent healthy tissues. For instance, Banerjee et al. employed a convolutional neural network (CNN) trained on MRI scans from 21 patients to differentiate between embryonal and alveolar subtypes of rhabdomyosarcoma, achieving an accuracy of 85% [32]. Similarly, Liu et al. developed a machine learning model leveraging radiomic features extracted from T2-weighted MRI to diagnose ileal Crohn’s disease. By integrating clinical and imaging data, their ensemble approach outperformed expert radiologists, reaching an AUC of 0.98 and an accuracy of 93.5% [33]. In another application, Wilson et al. introduced a computer vision–based algorithm to estimate small intestine length from magnetic resonance enterography (MRE) images in murine models, achieving a mean absolute error of just 1.8 ± 3.8 cm, providing a non-invasive alternative to traditional intraoperative measurement [34]. These applications showed the potential for generalizing CV models to various diseases.

#### Surgical navigation

CV enables real-time instrument tracking and spatial guidance during surgery. Souzaki et al. developed an AR navigation system that fused preoperative CT/MRI data with intraoperative imaging to guide pediatric tumor resections. Applied in six cases—including Wilms tumor and hepatoblastoma—the system facilitated complete resection in all patients and was particularly useful in visualizing small, otherwise undetectable tumors [35]. Additionally, Ward et al. created “POEMNet,” a deep-learning model trained on peroral endoscopic myotomy (POEM) procedure videos. The model accurately identified surgical phases with 87.6% precision, highlighting CV’s potential for workflow automation and intraoperative decision support [36].

#### 3D modeling for surgical planning

Advanced 3D modeling enhances anatomical understanding and preoperative planning. Gasior et al. evaluated how various imaging modalities—including 2D cloaca grams, 3D-CT reconstructions, interactive video models, and 3D-printed structures—impacted surgical comprehension in cloaca repair. Both trainees and attendings showed significantly improved performance with more immersive formats (*p* < 0.001) [37]. In another example, Lain et al. developed a noninvasive 3D scanning technique for assessing chest wall deformities, improving surgical planning in pectus excavatum repair by optimizing Nuss bar placement through Banana and Titanic indexes [38]. Similarly, Elkhill et al. demonstrated that combining CV with photogrammetry allowed for accurate 3D surface reconstruction of pediatric craniofacial deformities. These models helped streamline surgical planning and parent education while reducing reliance on CT imaging, thereby minimizing radiation exposure [39].

### Natural language processing (NLP)

Recent advancements in natural language processing (NLP) are increasingly impacting clinical practice by enhancing data extraction, pattern recognition, and decision-making processes across various medical specialties. From understanding patient perspectives to predicting clinical outcomes, NLP—often combined with ML—is proving invaluable in transforming unstructured data into actionable insights.

#### Patient-centered perspectives through social media analysis

Sollender et al. utilized NLP to analyze adolescent males’ perceptions of varicocele by examining discussions on a popular online forum. Focusing on users aged 21 and under, the study employed thematic analysis and the Meaning Extraction Method with Principal Component Analysis (MEM/PCA) to categorize conversation themes. Key topics included overviews of varicocele (27%), treatment strategies (19%), post-procedural experiences (19%), community support (17%), and second opinions (18%). More than half of the posts mentioned urologists, with varicocelectomy emerging as the most frequently discussed intervention. Notably, among adolescents reporting symptoms, pain (69%), cosmetic concerns (50%), and hypogonadism (27%) were commonly cited. These insights underscore the value of NLP in capturing real-world patient narratives, ultimately guiding clinicians in tailoring communication and care strategies to adolescent patients [40].

#### Enhancing cohort studies and longitudinal analysis

Kurowski et al. leveraged both codified data and NLP-derived variables to construct the largest North American single-center electronic medical record (EMR) cohort of pediatric- and adult-onset Crohn’s disease (CD) patients. Their analysis demonstrated that prolonged biologic therapy was associated with significantly reduced abdominal surgery rates. Adult-onset CD patients had higher 10-year surgery rates compared to pediatric-onset cases despite higher biologic use in pediatrics. Furthermore, treatment durations under six months were linked with increased surgical intervention rates across both groups. This study illustrates how NLP can facilitate large-scale, retrospective analysis of clinical narratives and enhance the depth of epidemiological research [41].

## Discussion

**Fig. 1:**
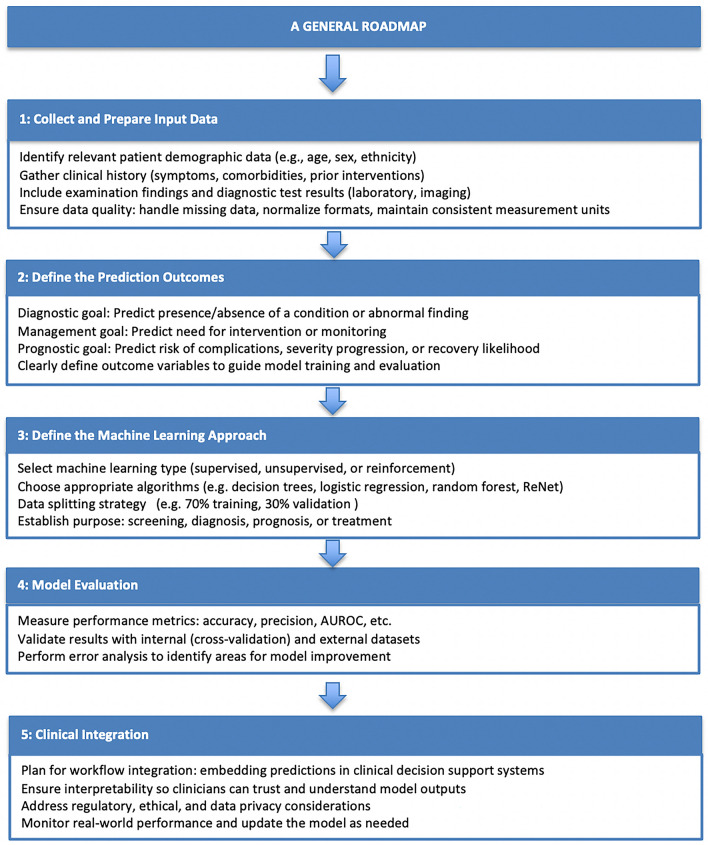
A structured roadmap outlining the key phases for implementing machine learning techniques in the clinical prediction of different diseases, emphasizing data acquisition, model development, evaluation, and integration into healthcare workflow

Many researchers emphasize that AI solutions in the medical field, particularly in pediatric surgery, are not designed to replace a doctor’s expertise but to complement it. The role of AI in healthcare is that of a powerful assistant, capable of supporting clinicians by providing valuable insights derived from real-time data analysis. The potential of AI does not lie in surpassing human judgment, but rather in enhancing decision-making process, especially in the high-pressure environment of pediatric surgery, AI can analyze large volumes of data at speeds and levels of precision that are simply unattainable for humans, thereby supporting early detection of issues and facilitating prompt adjustments in clinical decisions [1, 2]. As outlined in the results section, our comprehensive review of AI applications in pediatric surgery highlights a wide range of tools that have shown promise in improving patient outcomes. Among these, ML has emerged as the most impactful approach, offering significant potential in diagnostic support, risk stratification, and surgical outcome prediction, in various clinical scenarios, such as diagnosing appendicitis, and Hirschsprung disease or also monitoring conditions like necrotizing enterocolitis. Advancements in ML have contributed to more precise risk stratification, such as predicting complications and tailoring treatment options based on individual patient data [3]. These predictive capabilities enable clinicians to allocate resources more effectively, ensuring that high-risk patients receive appropriate interventions in a timely manner. By analyzing and breaking down the methodologies employed by various authors across different machine learning approaches, we created a structured roadmap that delineates the essential phases for applying ML techniques to clinical disease prediction. This roadmap highlights key stages—including data acquisition, model development, validation and evaluation, and integration into clinical workflows—and is intended to serve as a practical, adaptable guide for implementation in diverse healthcare settings (Fig. 1).

The integration of computer vision into surgical practice represents a transformative advancement, offering real-time assistance during operations and enhancing surgical precision [41]. For example, CV can be used to highlight critical anatomical structures, thereby supporting surgeons in performing procedures more accurately and reducing the risk of complications. This application of CV is already well established in adult oncologic surgery, as demonstrated in a recent review [42]. Specifically, CV has proven valuable in classifying polyps during colonoscopy and in landmark detection during laparoscopic surgery, as shown in studies involving adult patients [43, 44]. Looking ahead, as laparoscopic surgery for pediatric patients with solid tumors becomes more widespread, the use of CV in this context may represent a promising area for future research. Furthermore, AI-powered systems can contribute to post-operative analysis by providing feedback to refine surgical techniques and improve outcomes. Such advancements are particularly crucial in pediatric surgery, where visual data play a central role in both preoperative planning and intraoperative guidance.

However, despite the clear potential of such technologies, little attention has been paid to the practical challenges of implementation—namely, the significant investments required in terms of resources, time, and training. These barriers are further amplified in pediatric surgery, a field characterized by substantial variability in patient anatomy, case volume, and institutional expertise. As a result, tailored strategies will be essential to ensure that the benefits of computer vision and AI can be effectively translated into pediatric surgical practice (Table 2).

**Table 2:** Underexplored AI applications in pediatric surgery compared to adult surgery

Area of application	Status in adult surgery	Status in pediatric surgery	Challenge
AI-guided preoperative risk stratification	Widely used (e.g., LOS prediction)	Limited tools; rare data registries and small cohorts	Lack of large pediatric datasets; condition heterogeneity
AI-Enhanced Surgical Robotics and Autonomy	Limited clinical use used in urology, colorectal with AI-driven tasks	Minimal use	Size constraints, regulatory barriers, lack of pediatric-specific platforms
AI for Intraoperative Decision Support (e.g., computer vision)	Experimental phase (e.g., structure recognition)	Largely unexplored; no pediatric datasets or validated tools	Few annotated surgical videos; case rarity
AI in Postoperative Complication Prediction/Long-term outcomes	Widely used to predict infection, readmission, bleeding risks, QoL	Limited tools; mostly in research phase	Lack of integrated perioperative data systems for children
NLP for Operative Notes and Clinical Documentation	Used for quality control, adverse event detection, auto-documentation	Rare use in pediatrics; models not adapted to pediatric language	Need for pediatric-specific ontologies
AI in Surgical Education and Simulation	AI-enhanced simulators, skill tracking, rare case training available	Very limited; few pediatric-specific simulators with AI	Case complexity, limited training datasets

Natural Language Processing also holds significant promise in pediatric surgery. By analyzing unstructured clinical data, NLP enables the extraction of valuable insights from patient records, allowing clinicians to make informed decisions based on a comprehensive understanding of a patient’s history [45]. Despite the promising developments, the application of AI in pediatric surgery encounters several limitations. Many current studies are limited by small sample sizes, single-center evaluations, and a lack of prospective design. These limitations reduce the generalizability of findings and hinder the widespread adoption of AI tools in diverse clinical settings. One major challenge is the scarcity and quality of medical data. While the importance of large datasets is well recognized, the effectiveness of machine learning approaches depends not just on volume, but also on data quality—clean, well-labeled, and representative data. In pediatric surgery, where diseases are often rare and patient populations small, collecting such data poses a significant hurdle. To mitigate this, data augmentation methods—especially for histological analysis—have been introduced, yet ensuring reproducibility across similar datasets remains unresolved [10].

While the potential of AI in pediatric surgery is considerable, it is equally important to acknowledge its environmental impact. The training and deployment of large-scale AI models, particularly those relying on machine learning and natural language processing, require substantial computational resources and energy consumption. Data centers supporting these processes contribute significantly to carbon emissions, water use, and electronic waste generation [46]. This environmental burden raises ethical considerations regarding the sustainability of AI integration in medicine. In pediatric surgery—a field where innovation is often pursued to improve outcomes for vulnerable patients—it is crucial to ensure that technological advancements align not only with clinical priorities but also with broader commitments to environmental responsibility. Notably, the relatively small patient population in pediatric surgery suggests that the absolute environmental impact of AI adoption may be lower than in other specialties, and this footprint could be further offset by the healthcare resource savings gained through more accurate patient stratification and optimal allocation of care enabled by AI.

Future strategies may involve optimizing algorithms for efficiency, adopting greener computing infrastructures, and incorporating environmental impact assessments into the evaluation of new AI-based systems. By addressing these challenges early, the pediatric surgical community can promote responsible innovation that balances clinical benefit with ecological sustainability.

The lack of multi-institutional collaboration further impedes progress. While cross-institutional partnerships could enable the creation of large and diverse datasets, privacy regulations and concerns about healthcare data security pose formidable obstacles. In this context, federated learning offers a promising solution by allowing machine learning models to be trained across decentralized data sources without sharing sensitive patient data [47, 48]. However, this technique is still in early stages of adoption and faces logistical and technical barriers. Ethical concerns also persist. The use of AI in pediatric care raises questions about accountability, informed consent, and potential biases within algorithms. Because these tools are often seen as “black boxes,” their decision-making processes can lack transparency—making it difficult for clinicians and patients to trust or challenge AI-generated recommendations [49]. Ensuring that AI models are explainable, ethically developed, and validated through rigorous clinical testing is essential for their safe and effective implementation.

This review has some limitations. First, the rapidly evolving nature of AI technologies means that some of the applications and models discussed may soon be outdated, as new algorithms and approaches continue to emerge. Second, although we aimed to provide a comprehensive overview, many of the available reports are single-center based studies, with small sample sizes and limited external validation, which reduces the generalizability of their findings. Third, given the heterogeneity of study designs, methodologies, and outcome measures, a systematic comparison across different AI tools was not feasible, and the conclusions drawn should be interpreted with caution. Finally, our review was restricted to English-language publications and excluded conference abstracts, editorials, and commentaries, which may have limited the comprehensiveness of the evidence captured. While this approach ensured consistency in quality assessment and allowed focus on peer-reviewed full-length articles with sufficient methodological detail, relevant insights from non-English literature or preliminary findings presented in abstracts may have been missed.

## Conclusion

Artificial intelligence represents a transformative advancement in pediatric surgery, embodying the principles of hybrid intelligence. Its potential reaches far beyond feasibility; AI offers powerful tools to enhance diagnostic accuracy, anticipate disease progression, and personalize surgical care. These capabilities are especially valuable in pediatric populations, where clinical scenarios often involve considerable complexity and the rarity of conditions limits both clinical expertise and large-scale prospective research. In this context, well-conducted retrospective studies can also play a crucial role in generating meaningful insights and training robust AI models. However, pediatric surgery has historically lagged behind other specialties in the adoption of technological innovations, often attracting less industrial investment and fewer dedicated resources. As a result, the field must now actively engage with emerging digital tools and learn to speak the 'languages' of AI and data science to avoid falling further behind. Bridging this gap is essential to ensure that children benefit equally from the digital transformation reshaping healthcare. At the same time, the successful and responsible integration of AI into pediatric surgical practice requires careful navigation of key ethical considerations. Safeguarding patient data privacy, securing informed consent, and establishing robust regulatory frameworks are not optional—they are foundational to ensuring that AI technologies are implemented not only effectively but also transparently and ethically. By addressing these challenges, AI can be leveraged responsibly to improve clinical outcomes, reduce disparities in care, and ultimately advance the standard of pediatric surgical healthcare.

## Data Availability

No datasets were generated or analysed during the current study.
